# Investigation of The Effects of Prosthetic Knee Condition for Individuals with Transfemoral Amputation During Attempted Running

**DOI:** 10.33137/cpoj.v3i2.34481

**Published:** 2020-09-16

**Authors:** N. Blakeley, B. Silver-Thorn, J.A. Cross

**Affiliations:** 1 Department of Biomedical Engineering, Marquette University, Milwaukee, USA.; 2 Department of Orthopaedic Surgery, Medical College of Wisconsin, Milwaukee, USA.

**Keywords:** Kinematic, Gait analysis, Amputation, Prosthesis, Running, Energy efficiency, Gait symmetry, Prosthetic knee, Lower limb amputation

## Abstract

**BACKGROUND:**

A number of individuals with unilateral transfemoral amputation (TFA) run in a prosthesis with an unlocked prosthetic knee, while others choose to run with a locked prosthetic knee to increase stability. Research regarding running with an unlocked knee (UK) versus a locked knee (LK), with respect to energy efficiency, is limited and might be enhanced by characterization of the impact of knee condition on kinematics.

**OBJECTIVES:**

To investigate the effect of an UK versus LK on hip kinematics, energy efficiency, and running speed.

**METHODOLOGY:**

Five male novice runners with unilateral TFA completed one three-minute self-selected running speed (SSRS) trial and three peak speed trials per knee condition. Hip kinematics, energy efficiency, and running speed were compared between conditions.

**FINDINGS:**

Four of the five subjects exhibited a fast walk, rather than a consistent run. Hip flexion increased for all subjects and hip abduction decreased for four subjects during swing phase for the UK condition. Hip kinematic asymmetry was reduced for the UK condition in the sagittal plane for four individuals; hip kinematic asymmetry was also reduced in the frontal plane for the UK condition for three of these individuals. Mean energy efficiency was better for the UK condition (UK: 0.282 mLO2/kg/m, LK: 0.328 mLO2/kg/m). Peak running speed did not differ significantly between knee conditions (UK: 1.47m/s, LK:1.32m/s).

**CONCLUSIONS:**

For novice recreational runners with unilateral transfemoral amputation, the UK condition resulted in improved energy efficiency and enhanced kinematic symmetry, despite comparable peak speed relative to the LK condition. Therefore the UK condition may be advantageous for mid-range distance running.

## INTRODUCTION

When using a running specific prosthesis (RSP) at fixed speeds on a treadmill, individuals with a transfemoral amputation (TFA) consume 45-78% more oxygen than age-matched able-bodied runners.^1^ Some individuals with TFA run in a prosthesis with an unlocked prosthetic knee, others choose to run without a prosthetic knee; their prosthetic socket and foot are linked via a non-articulating pylon (no-knee condition).^2,3^ This non-articulating prosthesis will not buckle and collapse, regardless of load or runner fatigue, minimizing fall risk.^3^ Consequently, distance runners with TFA have reported decreased cognitive effort for the no-knee condition.3 The lack of a knee joint, however, requires that the individuals with TFA circumduct their prosthetic limb to clear the ground during swing phase.^4^ Leg circumduction during swing shifts the center of gravity laterally, thus decreasing energy efficiency.^5^

The effect of knee condition on oxygen consumption and running speed has been minimally investigated and results to date have been contradictory. For two experienced runners with TFA wearing RSPs, Wening and Stockwell reported that running without a prosthetic knee (no-knee condition) is more efficient based on level treadmill running trials at progressively increasing speeds.^3^ Both subjects ran for a prolonged period and achieved faster peak speeds when running in the no-knee condition. One subject demonstrated reduced peak VO2 and a faster speed for the no-knee condition, suggesting decreased energy efficiency. The other subject achieved a faster speed, but with an increased peak VO2, for the no-knee condition. Although Wening and Stockwell reported the no-knee condition was more energy efficient, as running speed was not controlled, this finding is inconclusive. In contrast, Highsmith et al. who tested four experienced runners with TFA reported that the unlocked knee (UK) condition was more energy efficient than the no-knee condition.^2^ Both self-selected running speed (SSRS) and peak speed tended to increase for the UK condition, although not significantly.^2^ However, mean oxygen consumption was only reduced for five of eight running speeds for the UK condition and prosthesis mass was not controlled. The potential variation in oxygen consumption with knee condition for runners with unilateral TFA may be related to hip kinematics as circumduction and increased hip abduction decrease energy efficiency due to the lateral shift in the center of gravity.^5^ However, hip kinematics for individuals with unilateral TFA running with a RSP have not been reported to date.

The goal of this study was to investigate whether a prosthetic knee unit should be unlocked or locked for individuals with unilateral TFA during recreational running on a treadmill based on hip kinematics, energy efficiency, and running speed. Running with a locked prosthetic knee is anticipated to introduce circumduction of the prosthetic limb for ground clearance. For the two knee conditions, the following results are anticipated: **1)** increased prosthetic limb hip flexion and reduced hip abduction during swing when running in the UK condition, **2)** reduced frontal and sagittal plane interlimb asymmetry of the hip during swing phase when running in the UK condition, **3)** better energy efficiency when running with the UK, and **4)** faster peak running speed for the UK condition. The population of TFA who currently run is small. To increase the sample size, our target population was novice runners, TFAs interested in extending their exercise regimen and potential fitness level, and trialing a running prosthesis. These individuals and their prosthetists might benefit from these trials and the related objective data acquired.

## METHODOLOGY

Five male subjects participated in the study (Table 1). Subject inclusion criteria were: unilateral transfemoral amputation, K3 to K4 activity level, 18-65 years, good general health, novice runners or individuals with interest and capability (as assessed by their physician or prosthetist), and body weight less than 100 kg (weight limit for the running prosthesis^6^). Individuals with balance disorders or neurological conditions that would adversely impact running, residual limb skin breakdown, or elevated vacuum suspension (incompatible with RSP) were excluded. The study protocol was approved by the affiliated Institutional Review Board (approval number HR-3249) and written informed consent was solicited and obtained for each subject prior to study participation.

**Table 1:** Subject characteristics and running experience.

	Subject 1	Subject 2	Subject 3	Subject 4	Subject 5	Mean (SD)
**Age (years)**	59	52	57	54	56	55.6 (2.42)
**Mass (without prosthesis, kg)**	72.8	93.0	87.4	82.5	93.4	85.2 (8.54)
**Height (with RSP, cm)**	184.0	186.0	178.0	185.5	176.5	182 (3.96)
Residual Limb Length^*^ (cm)	21	16	38	31	39	29 (9.14)
**Amputated Side**	Right	Left	Left	Right	Left	-
**Cause of Amputation**	Cancer	Cancer	Trauma	Trauma	Trauma	-
**Time Post Amputation (years)**	49	47	5	6	6	22.6 (20.75)
**Running Experience Everyday Prosthesis**	Short sprints during various sports (volleyball, tennis, basketball) within 1 month of testing, knee unlocked	None	None	None	Weekly fast walk 1.5-2 miles, on treadmill within 1 month of testing, knee unlocked	-
**Everyday Prosthetic Componentry**	**Suspension:** Suction **Knee:** Genium **Foot:** Triton Vertical Shock	**Suspension:** Suction **Knee:** X3 **Foot:** Triton Vertical Shock	**Suspension:** Suction **Knee:** C-Leg **Foot:** Triton 1C60	**Suspension:** Elevated Vacuum **Knee:** X3**Foot:** Triton Harmony	**Suspension:** Suction **Knee:** Genium **Foot:** Trias Plus	-
**Running Experience with RSP**	For the 2 weeks prior to testing, over ground running with knee unlocked and locked	One day per year, over ground with knee unlocked and locked	For the 3 months prior to testing, 3-4x per week on AlterG anti-gravity treadmill with knee unlocked	For the 3 months prior to testing, over ground with knee locked	None	-

* Distance from the greater trochanter to the most distal point on the femur (as determined by palpation).

An Ottobock (Duderstadt Germany) RSP consisting of the prosthetic socket, the 3S80 Modular Sport Knee Joint and either the IE90 Springlite Sprinter Foot (subjects 1, 2 and 4) or the IE91 Runner Foot (subjects 3 and 5) was used by all subjects. Each subject retained their original socket for both knee conditions. For subjects who did not own the Ottobock RSP, a fitting session was conducted by a collaborating certified prosthetist. The manual lock of the 3S80 knee was either locked (LK) or unlocked (UK), depending on the knee condition.

Due to time constraints (participant, prosthetist, laboratory, and loaned RSP's), a single training session (60-90 min) was conducted by the investigator to familiarize the subject with the equipment, test environment, and protocol as well as to determine the subject's SSRS in the UK and LK conditions. The treadmill training session included walking in both knee conditions, gradually progressing to a run at a pace dictated by the subject. Running at a steady-state speed was practiced for 1 to 3 minutes. Peak speed trials in each knee condition were conducted to familiarize the subject with the protocol. Once the subject verbally acknowledged they felt comfortable running in both knee conditions, the training session was concluded. A minimum of 72 hours post training, a single 3-4 hour testing session was conducted.

Running trials were conducted on an instrumented split-belt treadmill (Woodway, Waukesha, WI) while subjects were secured in a fall-arrest, safety harness. Subjects completed a warm-up period; the specific duration and activities (i.e., UK or LK, walking or running) were at the discretion of the subject. The warm-up period included a confirmatory determination of SSRS. The SSRS from the testing session was within 0.13 m/s of the training session SSRS for all subjects. Two three-minute running trials at SSRS were conducted (one per knee condition) followed by six peak speed running trials (three per knee condition) with a minimum of ten minutes rest between trials.^7^ Subjects were permitted a rest duration greater than 10 minutes, but all declined the extended time. For the three-minute running trials at SSRS, the treadmill was accelerated from rest to a comfortable walking speed and then increased to the subject's SSRS for the respective knee condition at a rate dictated by the subject.

The peak speed trials commenced in a similar manner after the SSRS was achieved: the speed was increased by 0.089 m/s every 3 seconds until the subject indicated they wanted to stop, pressed the emergency stop button, or engaged the safety harness.

**Figure 1:**
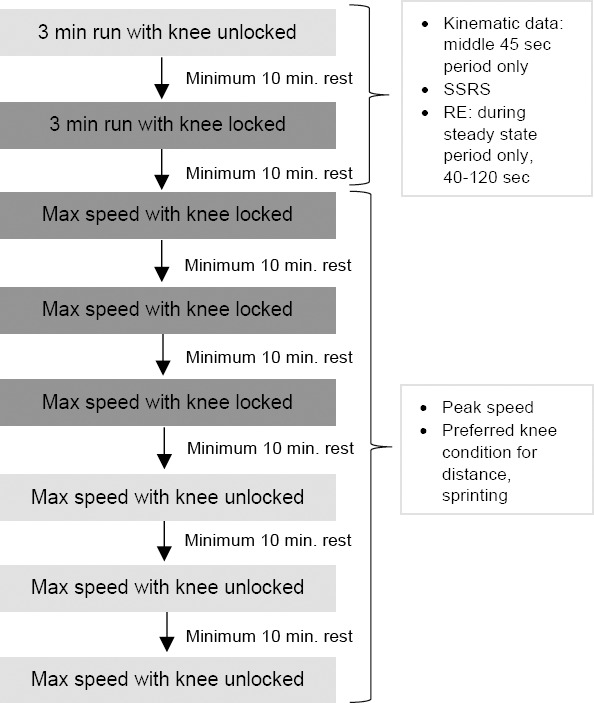
Flow chart overview of the running trials for the test protocol.

To minimize knee condition changes, the running trial order was not randomized. Switching knee conditions repeatedly would have increased the subjects' time burden, and randomizing the knee condition test order may adversely affect the subject's confidence. The UK condition was tested first as subjects routinely walk with an unlocked knee, thereby increasing their initial confidence and security during testing. Additionally, Highsmith et al. found the UK knee was more energy efficient,^2^ thus the UK condition sprints were completed last. The test order was SSRS UK, SSRS LK, peak speed LK, followed by peak speed UK (Figure 1).

Three-minute running trials were conducted first to decrease the effect of fatigue on metabolic results. Three peak speed trials with the knee locked were completed prior to peak speed testing in the UK condition, as the subject had just completed sub-maximal running with this knee condition. Peak speed was determined by accelerating the treadmill from rest to a comfortable walking speed and then increasing speed to the subject's SSRS for the associated knee condition. The speed then increased by 0.089 m/s every 3 seconds until the subject indicated they wanted to stop, pressed the emergency stop button, or engaged the safety harness. The final speed achieved prior to the occurrence of one of these events was recorded as the trial's peak speed. At the conclusion of the testing session, subjects were asked to identify the preferred knee condition (UK or LK) for distance running and sprinting.

**Figure 2:**
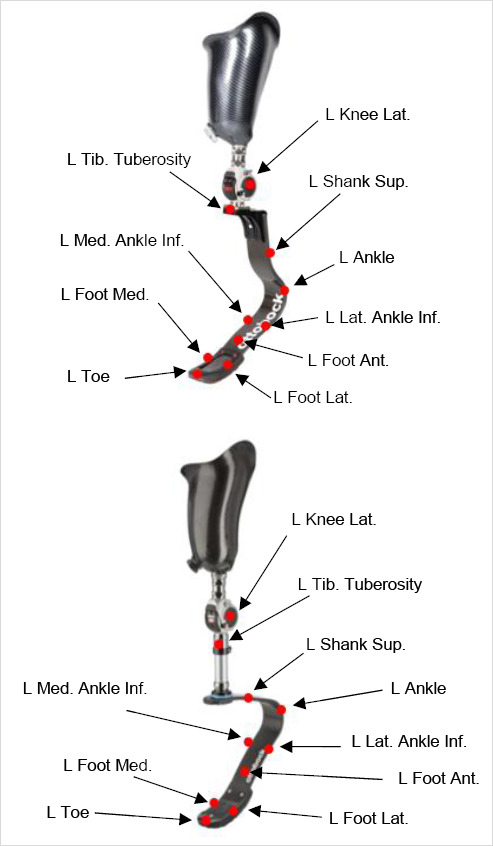
Marker placements on the 1E90 Sprinter foot (Top) and 1E91 Runner foot (Bottom) (Figure adapted from Ottobock^11,12^). Not pictured: “heel” marker placed posteriorly at the most acute radius of the foot.

A 13-camera motion capture system (OptiTrack, Corvallis, OR) was used to acquire kinematic data (120 Hz, low pass filtered with a fourth order Butterworth, cut-off frequency of 6 Hz ^8,9^) during the three-minute running trials. The filter cut-off frequency was determined based on a fast Fourier transform of the left and right heel and anterior superior iliac spine marker position data. Thirty five retro-reflective markers were secured to the subject based on a conventional gait model, modified for the prosthetic limb,^4,10^ using Visual 3D software (version 6, C-Motion, Germantown, MD). Consistent with the conventional gait model, the hip angle was defined as the thigh relative to the pelvis. Markers were placed on the sacrum and bilaterally positioned on the iliac crest, anterior superior iliac spine, greater trochanter, lateral femoral epicondyle, medial femoral epicondyle, tibial tuberosity, lateral malleolus, medial malleolus, fifth metatarsal, second metatarsal, and calcaneus. Marker placements on the RSP are shown in Figure 2. A static trial was conducted for each subject and knee condition to define the local coordinate systems.

To determine stride cycle events, vertical force data were collected (1200 Hz) from the instrumented split belt treadmill, low pass filtered (zero phase 8th order Butterworth filter, cutoff of 12 Hz^13^), and down sampled to match the sampling frequency for the kinematic data; an amplitude threshold of 44.5 N was implemented to define heel strike (HS) and toe-off (TO) events.^14^ A custom MATLAB (version: 9.1.0.441655, The MathWorks, Inc., Natick, MA) script was written for vertical force data processing.

Specific kinematic parameters, extracted bilaterally for analysis, included peak hip flexion and peak hip abduction during swing phase. To assess the asymmetry of these kinematic parameters between the prosthetic and intact limbs during each knee condition, the interlimb asymmetry (IA) index (Equation 1)^15^ was calculated using Microsoft Excel (Version 1908, Microsoft Corporation, Redmond, WA) for discrete stride cycles in the middle 45-second period of the three-minute SSRS trial. Stride cycles were excluded if marker drop-out exceeded 10 frames and affected the calculated kinematic parameter of interest. For a given subject and kinematic parameter, the number of stride cycles (6-17 cycles) retained for analysis was the same between knee conditions, randomly omitting the extra stride cycles for the knee condition with more cycles. For each subject and knee condition, the mean and standard deviations were calculated across all included stride cycles in the analyzed time period for IA for peak hip flexion and abduction during swing phase, respectively.


Equation 1: $$IA=\left(\frac{X_{\text{intact}}-X_{\text{prosthetic}}}{X_{\text{intact}}+X_{\text{prosthetic}}}\right)*100\%$$

X_prosthetic_ and X_intact_ represent the specific kinematic measures (peak swing phase hip flexion and abduction) for the prosthetic and intact limbs, respectively. An IA index value of 0 represents symmetry; negative IA values indicate that the parameter value for the prosthetic limb exceeded that for the intact limb. The percentage of asymmetry is reflected by the IA magnitude (e.g. an IA index of -20 and +20 represent the same magnitude of asymmetry).

VO2 measurements were collected breath-by-breath using the K4b2 portable metabolic system (Cosmed USA Inc, Chicago IL) during the full three-minute trials for both knee conditions. VO2 data were averaged over 20 second intervals for the entire data series. Running economy (RE), a measure of energy efficiency during running, was calculated from the steady-state VO2 portion of the trial. The steady-state portion of the VO2 was defined as a change in VO2 of less than 100 mL/min.^16^ Based on this definition, all participants achieved steady-state for both knee conditions with durations ranging from 40 to 120 seconds. RE was calculated as the ratio of the body-mass normalized steady-state VO2 to the SSRS for the corresponding three-minute trial. RE was calculated both inclusive and exclusive of prosthesis mass.

### Statistical analyses

Statistical analyses were conducted using SPSS (v 24.0, IBM Inc., Armonk, NY). The Shapiro-Wilk test (p=0.05) was used to assess data normality for all variables. The UK condition was anticipated to reduce hip kinematic pathologies during swing. Swing phase peak prosthetic limb hip flexion, abduction, and hip frontal and sagittal plane interlimb asymmetry were tested with two-tailed paired t-tests (p=0.05, if normally distributed data) or the Wilcoxon signed-rank test (p=0.05, if non-normally distributed data). All kinematic variables were assessed on a single-subject basis in a separate test for each knee condition, using all retained stride cycles. Thus, there were 6-17 data points per statistical test. The corresponding effect size was calculated using Cohen's d (normally distributed data) and Equation 2 (non-normally distributed data):


Equation 2: $$r=Z/\sqrt{N}$$

where r represents effect size, Z represents the test statistic, and N represents the number of samples.

In contrast to the kinematic parameters, single values of RE and peak speed were available for each knee condition and subject. These data were assessed on an inter-subject basis using a paired t-test (p=0.05). These group statistics, while statistically significant, are referred to as “trends” due to the small sample size.

## RESULTS

Due to the novice running status of the recruited subjects, only subject 1 was able to run (i.e. exhibit periods where both feet were airborne simultaneously) consistently throughout the SSRS and peak running speed trials. Subjects 2-5 all exhibited a fast walk. Subjects 1 and 2 did not require the use of the handrails while subjects 3-5 used the handrails consistently throughout all the trials for both knee conditions. The UK condition was preferred for all subjects for distance running and subjects 2 and 4 preferred the LK condition for sprinting.

### Kinematics:

**Figure 3:**
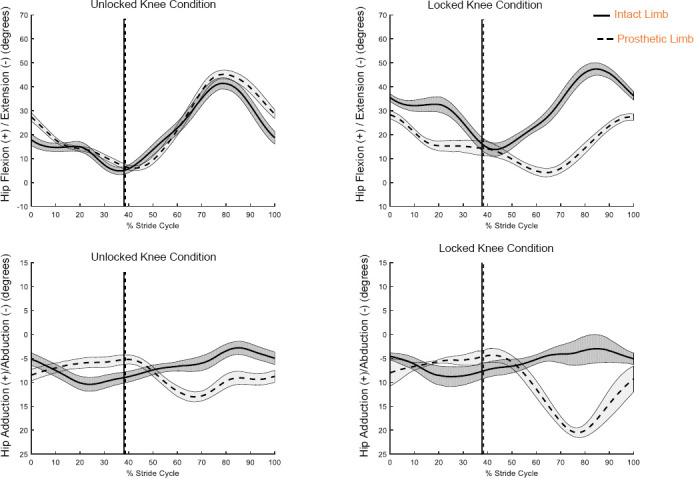
Mean (middle 45 seconds) hip motion in the sagittal (Top) and frontal planes (Bottom) for Subject 1 during the self-selected running speed trial in the unlocked (left) and locked (right) knee conditions. Vertical lines denote toe-off.

Statistically significant single-subject differences in maximum hip flexion during swing were found between knee conditions across all subjects. Peak hip flexion of the prosthetic limb increased during swing with the UK condition for all subjects (Table 2, Figure 3, see Nelson^17^ for all kinematic graphs). A statistically significant difference in peak IA for the hip in the sagittal plane between knee conditions was found for each subject (Table 2).

**Table 2:** Sagittal plane hip kinematics (mean (SD)) during swing phase for select strides in the middle 45 seconds of the three-minute self-selected running speed trial for each subject for each knee condition. **Bold** values denote statistically significant single-subject differences (0.05 level) between knee conditions.

	Subject 1		Subject 2		Subject 3		Subject 4		Subject 5	
	UK	LK	UK	LK	UK	LK	UK	LK	UK	LK
Prosthetic Limb Peak Hip Flexion (°)	**45.7 (1.05)**	**27.4 (1.34)**	**48.2 (2.72)**	**31.8 (2.22)**	**60.3 (2.03)**	**57.2 (4.25)**	**53.5 (1.50)**	**40.6 (1.76)**	**53.8 (1.57)**	**51.8 (1.72)**
	**^+^ *P* <0.001**, effect size: 10.80 [6 cycles]		**^+^ *P* <0.001**, effect size: 4.29 [7 cycles]		**^+^ *P =*0.012**, effect size: 0.99 [10 cycles]		**^+^ *P* <0.001**, effect size: 6.52 [17 cycles]		**^+^ *P =* 0.001**, effect size:0.26 [17 cycles]	
IA for peak hip flexion (%)	**-2.87 (1.35)**	**25.90 (2.54)**	**-4.58 (2.49)**	**16.40 (4.10)**	**0.29 (2.50)**	**9.29 (3.73)**	**-3.58 (1.67)**	**9.10 (2.37)**	**-2.10 (1.95)**	**0.65 (2.10)**
	^*^ *P =* 0.001, effect size:1.34 [6 cycles]		**^+^ *P* <0.001**, effect size:3.36 [7 cycles]		**^+^ *P =* 0.006**, effect size:1.84 [6 cycles]		**^+^ *P* <0.001**, effect size:4.62 [15 cycles]		**^+^ *P =* 0.001**, effect size:1.09 [15 cycles]	

+ Denotes a two-tailed paired t-test was conducted (normally distributed data).

* Denotes the Wilcoxon signed-rank test was conducted (non-normally distributed data). UK = Unlocked knee, LK = Locked Knee

For four subjects (1-4), the prosthetic hip abduction during swing increased (greater negative angle) from the UK condition to the LK condition (Table 3, Figure 3, see Nelson^17^ for all kinematic graphs). For all subjects, peak IA for the hip in the frontal plane during swing differed significantly between knee conditions (Table 3); in terms of magnitude, IA decreased for three subjects (1, 2, and 4) for the UK condition, reflecting increased symmetry for this knee condition.

**Table 3:** Frontal plane hip kinematics (mean (SD)) during swing phase for select strides for the middle 45 seconds of the three-minute self-selected running speed trial for each subject for each knee condition. **Bold** values denote statistically significant *single-subject* differences (0.05 level) between knee conditions.

	Subject 1		Subject 2		Subject 3		Subject 4		Subject 5	
	UK	LK	UK	LK	UK	LK	UK	LK	UK	LK
Prosthetic Limb Peak Hip Abduction (°)	**-13.2 (0.783)**	**-20.7 (0.968)**	**-7.5 (0.651)**	**-18.4 (1.27)**	**-15.4 (1.95)**	**-18.4 (2.63)**	**-5.5 (0.753)**	**-16.1 (1.38)**	**-15.5 (1.64)**	**-13.7 (1.48)**
	**^+^ *P* <0.001** effect size: 5.23		^*^ *P =* 0.018 effect size: 0.09		**^+^ *P =*0.017** effect size: 0.93		**^+^ *P* <0.001** effect size: 6.41		**^+^ *P* <0.001** effect size:1.18	
IA for peak hip abduction (%)	**-19.4 (4.53)**	**-43.6 (5.24)**	**14.8 (7.36)**	**-20.9 (8.93)**	**-83.0 (4.09)**	**-65.0 (4.30)**	**22.5 (6.52)**	**-27.3 (6.45)**	**-32.4 (9.73)**	**-15.6 (7.70)**
	**^+^ *P =* 0.002** effect size:2.39		^*^ *P =* 0.001 effect size:1.24		**^+^ *P =* 0.003** effect size:2.26		**^+^ *P* <0.001** effect size:5.35		^*^ *P* <0.001 effect size:1.40	

+ Denotes a two-tailed paired t-test was conducted (normally distributed data).

* Denotes the Wilcoxon signed-rank test was conducted (non-normally distributed data).UK = Unlocked knee, LK = Locked Knee

### Energy Efficiency

A trend was found for differences in RE between knee conditions, regardless of whether prosthesis mass was included or excluded in the normalization. Mean RE values were reduced for the UK condition for the RE calculated exclusive of prosthesis mass (Table 4, see Nelson^17^ for RE normalized inclusive of prosthesis mass).

**Table 4:** Running economy during the three-minute SSRS trials; normalization is exclusive of prosthesis mass.

	Subject 1		Subject 2		Subject 3		Subject 4		Subject 5		Group Mean (SD)	
	UK	LK	UK	LK	UK	LK	UK	LK	UK	LK	UK	LK
RE(mLO_2_/kg/m)	0.301	0.348	0.290	0.286	0.266	0.305	0.332	0.430	0.223	0.270	**0.282 (0.037)**	**0.328 (0.057)**

### Running Speed

SSRS ranged from 0.890 to 1.79 m/s (mean SSRS: UK=1.47(SD=0.260) m/s, LK=1.32 (SD=0.246) m/s). All five subjects exhibited faster SSRS for the UK condition (Table 5). Peak running speed ranged from 2.15 to 3.30 m/s (average peak speed: UK=2.72 (SD=0.450) m/s, LK=2.61 (SD=0.320) m/s).

**Table 5:** RSSRS and peak speed (maximum value of three trials) for each knee condition.

	Subject 1		Subject 2		Subject 3		Subject 4		Subject 5		Group Mean (SD)	
	UK	LK	UK	LK	UK	LK	UK	LK	UK	LK	UK	LK
SSRS(m/s)	1.34	1.29	1.43	1.34	1.79	1.48	1.07	0.890	1.70	1.61	1.47(0.260)	1.32(0.246)
Peak Speed (m/s)	2.24	2.32	2.86	2.86	3.30	2.95	2.15	2.15	3.04	2.77	2.72 (0.450)	2.61 (0.320)

## DISCUSSION

Research regarding the effect of knee condition on lower extremity kinematics for individuals with unilateral TFA is limited; the observed kinematic data cannot be contrasted with the literature. For novice runners with unilateral TFA, LK running can be characterized by reduced peak hip flexion of the prosthetic limb and increased hip abduction during swing (Table 2, Table 3). The more extended and abducted prosthetic hip during swing is likely attributed to circumduction of the prosthetic limb to provide floor clearance. In contrast to the UK condition for which knee flexion assists with floor clearance in the sagittal plane, LK running requires both sagittal and frontal plane hip motion to provide clearance. Future work might include an analysis of the bilateral knee kinematics to provide more insight into how foot clearance is accomplished.

Although swing phase pelvic kinematic data were not presented, it was collected and can assist in the interpretation of the hip kinematic data. The pelvis was typically tilted anteriorly during running in the LK condition. For subjects 3-5, this may be attributed to their leaning on the handrails for support. Pelvic motion in the frontal plane was variable: subjects 1-3 exhibited ipsilateral tilt (pelvis tilted downwards on the prosthetic limb side) during LK running and subjects 4 and 5 exhibited contralateral tilt (pelvis tilted upwards on the prosthetic limb side) during LK running. All subjects exhibited external pelvic rotation (rotated away from center of body) on the prosthetic limb side. Specifically, subject 5 demonstrated approximately 17° of external pelvic rotation compared to 3°-11° external pelvic rotation for subjects 1-4.

Although all subjects exhibited a decrease in peak swing phase hip flexion during LK running, this difference was relatively small for subjects 3 and 5 (2-3°) relative to the other subjects (13-18°). During LK running, hip abduction of the prosthetic limb increased during the swing phase by 3° to 11° for subjects 1 and 4, potentially due to the circumduction of the prosthetic limb for floor clearance during LK running. It should be noted that because the hip angle was defined as the thigh relative to the pelvis, an increase in hip abduction may be attributed, at least in part, to upwards pelvic obliquity. Subject 5 did not exhibit an increase in swing phase hip abduction with the LK condition potentially due to adopting a different strategy to clear the prosthetic limb as he was one of the least experienced runners with an RSP. The unanticipated decrease in hip abduction with the LK condition may have been inadvertently masked by defining the hip angle as the thigh relative to pelvis. For example, if the trunk and pelvis were leaning away from the swing limb to aid foot clearance, no increased hip abduction would be observed. The anticipated increase in prosthetic limb hip flexion during swing was not observed, nor was the anticipated reduced hip abduction during swing observed with the UK condition.

The UK condition resulted in better symmetry in peak hip flexion (maximum hip angle) for four subjects (1-4) as indicated by the reduced IA magnitude compared to the LK condition (Table 2).

In the frontal plane, IA differed significantly on a single-subject basis between knee conditions for peak hip abduction (minimum hip angle) during swing phase for all subjects. Three subjects (1, 2, 4) demonstrated improved symmetry during UK running. In contrast, subjects 3 and 5 exhibited improved frontal plane hip symmetry during swing for the LK condition. The anticipated reduced hip IA in the frontal and sagittal planes when running in the UK condition was therefore not observed. A possible explanation for this unanticipated result is that subjects 3 and 5 adopted an altered circumduction pattern. For subjects 1, 2, and 4, prosthetic limb hip flexion occurred approximately 13-22% stride cycle later than hip abduction. Subjects 3 and 5, however, initiated hip flexion and abduction simultaneously.

For subject 3, this manifested as increased hip flexion and reduced hip abduction during circumduction, leading to a narrower circumduction path. This may be due to his experience with UK running (Table 1) leading to a habit of pulling the prosthetic limb directly underneath his body, as opposed to circumduction. In contrast, subject 5 externally rotated his pelvis on the prosthetic limb side just prior to toe-off which may have facilitated the simultaneous hip flexion and abduction. This may be a technique to achieve circumduction with decreased hip abductor muscle activity on the intact limb.

It is important to note that the socket design was kept consistent between knee conditions for each subject; therefore, the socket design did not influence results on an individual subject basis, but it may have influenced results between subjects. Additionally, the length of the prosthesis was not adjusted when switching between knee conditions. A prosthetist may elect to shorten the prosthesis when leaving the knee locked to assist in limb clearance during swing phase. Thus, the observed increased hip abduction during LK running may be attributed in part to overall prosthesis length.

While not statistically significant, a trend was found for grouped-subject differences also observed in RE (Table 4) across subjects. Mean RE values were improved for the UK condition, indicating that for this population of novice runners with TFA, running with an UK was more efficient than running with a LK, as expected. These results are consistent with Highsmith et al.^2^ who observed reduced mean oxygen consumption for the UK condition for five of eight running speed stages (1.12-2.01 m/s). The decreased energy efficiency observed for the LK condition is likely attributed to the pathologic prosthetic limb circumduction strategy adopted to provide floor clearance. The improved RE with the three-minute running trial also supports the UK condition preference for distance running for all five subjects.

Finally, for the novice runners with unilateral TFA in this study, the average peak running speed was faster for the UK condition (unlocked: 2.72 ± 0.450 m/s, locked: 2.61 ± 0.320 m/s). Contrary to initial expectations, only two subjects (3 and 5) ran faster in the UK condition. The results were consistent with Highsmith et al.^2^ who also observed no significant difference in peak running speed with knee condition for runners with unilateral TFA. These findings, however, contradict Wening and Stockwell^3^ who reported faster speeds for the no-knee condition. Neither study, however, included statistical analyses as the investigations included just two and one subject, respectively. These previous studies tested experienced runners with unilateral TFA for a much longer duration (10-17 minutes^2,3^ versus 30-60 seconds). Their protocols therefore measured peak endurance speed rather than sprinting capacity.

Similarly, the mean SSRS for the UK (1.47 (SD=0.260) m/s) was faster than for the LK condition (1.32 (SD= 0.240) m/s). The faster SSRS for the UK condition may indicate the UK is advantageous for treadmill running, provided that the subject has the endurance and cognitive focus to prevent knee buckling. In contrast, Highsmith et al. did not find significant differences in SSRS between knee conditions.

### Limitations

A primary limitation of this study was the small sample size. Post hoc power analysis indicated the power associated with the peak speed parameter was only 16.8%. Given the lack of statistically significant differences in peak speed with knee condition, variations in test methodology between the current study and previous studies, definitive conclusions regarding which knee condition facilitates increased speed cannot be stated.

Another limitation is the small magnitude of observed differences in hip kinematics between knee conditions. While these differences were statistically significant, they are likely not clinically relevant. A 2°-3° difference in peak hip flexion (subject 5) and peak hip abduction (subjects 3 and 5) between knee conditions may have been imperceptible to the subject.

Similarly, the relatively short duration (three minutes) of the SSRS trials likely limits the potential clinical and/or real-world relevance of the study findings. While the differences in SSRS were modest, such differences may be relevant if sustained during increased duration running trials.

Lastly, subject 1 was the sole participant who actually “ran”, exhibiting periods during which both feet were airborne simultaneously. The ambulation of the remaining subjects might be more accurately described as a “fast walk”, with a few cycles of true running interspersed. Future protocols might incorporate more extensive training for both knee conditions, prior to data collection, to more effectively assess subjects' true running performance. Additionally, a second test session to determine repeatability of the novice runners' performance would have been ideal; however, time constraints did not permit this.

Clinical Recommendations: Running with a LK increases stability of the prosthesis, decreasing fall risk and cognitive load^3^; these factors are likely important during running for prolonged periods and longer distances. For recreational, short distance running on a treadmill, the results of this study suggest that the UK condition may be advantageous for novice runners with unilateral TFA. The LK condition resulted in decreased energy efficiency and a slower SSRS. The UK condition may also decrease risk of musculoskeletal injury, as this knee condition resulted in minimal gait pathologies. The LK condition required circumduction for floor clearance, a gait pathology that impacts hip kinematics in multiple planes and may also affect pelvic and trunk motion.

## CONCLUSION

Hip flexion decreased for all subjects and hip abduction increased for four subjects during swing when individuals with unilateral TFA ran with a LK due to compensatory circumduction to assist with foot clearance. This circumduction increased IA during swing for the peak hip flexion and peak hip abduction measures and may contribute to the decreased energy efficiency observed during LK running. Based on these results and the relatively short running duration in this study, the UK condition is recommended for novice runners with unilateral TFA when running short distances on a treadmill.

## DECLARATION OF CONFLICTING INTERESTS

The authors declare that there is no conflict of interest.

## AUTHOR CONTRIBUTION

**Natalie Blakeley:** designed the study, acquired the data, analyzed the data, interpreted the data, and drafted the manuscript.

**Barbara Silver-Thorn:** designed the study, interpreted the data, and revised the manuscript.

**Janelle A. Cross:** assisted in the design of the study, interpreted the data, and revised the manuscript.

## SOURCES OF SUPPORT

Clinical and Translational Science Institute Pilot Translational and Clinical Studies Program Start-up Project Award. This award is supported by grant UL1TR001436 from the Clinical and Translational Science Award (CTSA) program of the National Center for Research Resources and the National Center for Advancing Translational Sciences.

## ETHICAL APPROVAL

The study protocol was approved by the affiliated Institutional Review Board (approval number HR-3249) and written informed consent was solicited and obtained for each subject prior to study participation.
